# Transactions of the Association of Army and Navy Surgeons

**Published:** 1864-03

**Authors:** 


### TRANSACTIONS OF ASSOCIATION OF ARMY AND NAVY SURGEONS.

*Debate on Tetanus.*

Association of Army and Navy Surgeons, January 30th, 1864.

Surgeon-General S. P. MOORE, President, in the chair.

The President called the Association to order at seven, P. M, Surgeon DAVIS presented the minutes of the last meeting, which were read and approved.

Surgeon MICHEL read a letter from Surgeon E. S. GAILLARD, communicating information of a death from chloroform. The anæsthetic was inhaled about thirty seconds, when the patient was attacked with convulsions; the pupils were extraordinarily dilated, and the pulse suddenly ceased at the wrist. Artificial respiration and cold effusions were freely used. The autopsy disclosed extreme cerebral congestion. The heart was not examined. The same preparation of chloroform had been used with impunity.

A letter from Surgeon CHAMBLISS was also read, defining tetanus to be a general tonic contraction of all or most of the voluntary muscles, increased by paroxysms; while tetanic spasms are partial contractions of the muscles of a part. True tetanus is the disease in its most general, aggravated and advanced form; whilst tetanic spasms are the threatening premonitory or milder form. Of 1,241 cases of gun-shot wounds treated in the second division of the Winder hospital, but one case of true tetanus occurred, and that took place on the twelfth day after the injury.

Another letter read, from Surgeon THOM, defined tetanus as the fully-developed disease affecting the whole system, and tetanic spasms as confined to certain muscles of the extremity. Patients affected with the latter often recover, but, in his experience, traumatic tetanus was invariably fatal. He had seen in the last two years but three cases of tetanus and all died. The symptoms in one case occurred twelve days and in another nine days after injury.

From Surgeon HABERSHAM two interesting cases of tetanus from gun-shot wounds were communicated by letter. One a flesh wound, the other a compound fracture of fibula; both commencing with trismus and ending in general tetanus; one on the eleventh day, the other on the twelfth, from injury; the former terminated in death in a few hours, the latter in twenty-four hours.

Surgeon MICHEL now moved that the general treatment of gun-shot wounds be taken up for reconsideration, as not having been entirely disposed of at the last meeting.

The Chair considered that the subject had been already discussed and disposed of, and, on submitting the motion to the Association, it was overruled. The President then introduced, as the subject for debate, the differences between "Tetanus and Tetanic Spasms."

[It is to be regretted that the term *traumatic spasm* had not been employed in preparing the question submitted to the Society for discussion, rather than the more indefinite expression tetanic spasm; the former having been already adopted into the literature of the subject as a scientific expression, while the latter phrase is vague and requires explanation.—ED ]

Surgeon CAMPBELL said he would relate a case formerly under treatment, heretofore classed by him under the head of partial chronic tetanus, but which, perhaps, might be better termed one of "tetanic spasms." Some years ago, a negro man, wounded in the neck, at the explosion of a gun on board the United States ship Princeton, was brought to Augusta, Georgia, where, a year after the accident, he came under his surgical care. He was affected with partial convulsions of the muscles of the neck and back, and was relieved by the exhibition of sedatives. Patient stated that this was not his first attack; he had had "lock-jaw" shortly after the wound, and spells of such symptoms as constituted the present attack even since his recovery. The attacks returned at intervals of from two or three weeks to as many months. He administered two and a half grains of quinia and ten grains of precipitated carbonate of iron, three times a day, for several months. He was very willing to accept the term tetanic spasms as a proper designation to characterize it. The treatment had been suggested by the regularity of the relapses, simulating the course of the disease to the hebdomadal periods observed by intermittent fever and other paroxysmal diseases, confessedly so amenable to these means of tonic medication. With regard to the nature of tetanic spasms, he considered them as identical, in nature and pathology, with tetanus; the former being applied to tetanic phenomena affecting a restricted portion of the muscular system—the excito-motory arc being shorter—and the symptoms less acute in their character; while, in true tetanus, the entire muscular system was subjected to universal tension.

Surgeon McCAW observed, that he could not discern any essential difference between the local and general symptoms contemplated in the question before the society. He believed spasms of the kind referred to, developed in consequence of a wound, exhibited the disease in its mildest form. Any excitation of the peripheral nerves produces an excito-motory action, and, however limited in extent, it is the same phenomenon as disclosed in the more extensive contractions, sometimes of the whole body. For him, this was a distinction without a difference.

Surgeon MICHEL remarked, that he recognized an important difference between true tetanus and tetanic spasms, however difficult it might be either to define or explain the conditions upon which their dissimilarity depended. One fact was certain, for experience had taught it, that, while tetanus was justly regarded as the most rapidly fatal malady with which the surgeon has to contend, tetanic spasms yield to ordinary treatment, or to no treatment at all. If the convulsive movements be restricted to the injured limb, and we find them in nowise compromitting the general condition of the patient, we need entertain no decided misgivings as to his safety. He considered it necessary to draw some distinction between these morbid exhibitions in estimating the ratio of cures which might be recorded in the extensive statistics received from the army. Without discrimination in this particular, and without impressing the subject upon the mind, results of treatment and cures would be recorded which would be found strangely at variance with what is known regarding the mortality in this disease. His military surgery had furnished no case of tetanus, but in civil practice he had seen several. One of these would illustrate the point under discussion: a negro girl, with an oyster-shell wound of the foot, was seized with spasms in the same limb, which progressed until well-marked tetanic contractions of the part were developed; but at no time was there any trismus, and never did the spasms become general. The attack lasted two weeks. Chloroform not being then in use, opium in large doses was given with no improvement, until combined with musk, which he had been advised to try. The girl recovered; and at the time, a beginner in the profession, he was very sure he had cured a case of tetanus, and that musk was the remedy. Sometime after, alluding to the cure, a colleague informed him that the same girl had been a patient of his, and that he had attended her with spasms of a like character from the cut of a lash, which had scarcely left a mark. He believed true tetanus nearly always commences with trismus, and not in local contractions of the wounded limb.

Surgeon WILSON said he was ready to endorse Dr. CAMPBELL'S views of the pathology of tetanus; as, under any of the forms in which it declares itself, the pathology was always the same. These conditions were differences of degree, not of kind.

Surgeon McCAW asked if the gentleman would state what was the pathology of tetanus. Hysteria also was a reflex action of the nervous system. He had seen hysterical patients thrown into violent spasms, which assumed all the forms described under the names of opisthotonos, emprosthotonos, &c. He considered Dr. MICHEL'S case as possibly one of those hysterical cases simulating tetanus. Hysteria, as it were, was a mere functional derangement, though it was almost impossible at times to see any difference between it and tetanus, from the condition into which the patient is thrown. Tetanus, however, was an organic disease. If called upon for a prognosis in the spasms of which we were speaking, he supposed he should have to wait; and should the patient recover, he would say he had had "tetanic spasms;" but if he died, of course it was "tetanus."

Surgeon CAMPBELL replied, that having referred the two affections of tetanus and tetanic spasms, to a pathological condition which he thought identical, by no means indicated that he professed himself willing to state with confidence, precisely what that pathology is. He would, however, state, that in his opinion, tetanus was an exhibition of exalted reflex-motory action, dependent upon a morbid condition of the spinal marrow, rendering the spinal centres unusually susceptible to external impressions, and less obedient to the determinations of the will. Whether this abnormal condition of the spinal marrow was inflammatory in its character, or belonging to those less appreciable conditions termed molecular, the present state of our knowledge did not determine; but there are many observations on the dead subject which lead to the conclusion, that at least, in some instances, the inflammatory action at the wound is propagated along the course of the nerve leading from the wound, on to the point at which it is implanted into the spinal marrow. The neurilemma is often found intensely inflamed, and decided congestion is not uncommon both in the spinal marrow and its membranes. Corroboration of this pathology of tetanus is found in the fact, that, all the most successful plans of treatment are such as this view would suggest, namely: such measures as are best calculated to quiet or obtund both peripheral and centric irritation, as sedatives, anæsthetics and topical application of ice, to the spinal column. Tetanus was one kind of reflex action, requiring a peculiar condition of the organs acted upon, and probably a *peculiar* cause. Hysteric cases not only similated tetanus, but as far as identity of phenomena, organs involved, and rationale of production are concerned, they are indeed *true cases of tetanus*. Resulting in recovery, and being based on an hysteric condition of the nervous system, did not weaken the grounds for classing them among tetanic cases. We do not hesitate to call a case of deranged gastric digestion manifesting all the symptoms of dyspepsia, by that name, because it happens to arise from uterine derangement. Dr. C., also adduced, in illustration, a case of sudden and complete blindness, lasting for weeks, which resulted from menstrual obstruction. This case, he said, no one would hesitate to call amaurosis, on account of its having arisen from uterine derangement, and no more should we be unwilling to designate the case described by Dr. MICHEL as tetanus, because it happened to be based in hysteria. He considered the phenomena identical in both cases, notwithstanding the diversity of cause and prognosis.

Surgeon MICHEL rose to say, that it was evident that all morbid manifestations, dependent upon perverted nervous action, must exhibit resemblances so striking as to place them, with every propriety, in one class, since preternatural exaltation of the nervous centres could display itself only by excito-motory or secretory acts. It was important, however, not to confound such phenomena, amidst all the possible variations they present, under one generic appellation—such as tetanus, for example—because the same apparatus was involved in their production, or because the physiological explanation seemed to be the same. Excitor impressions, if continued long enough to evoke centric action, would, of course, result in the evolution of inco-ordinate muscular contractions, which, strangely interesting to state, always presented peculiarities so manifest as to permit the recognition of very constant differences in these exhibitions of reflex function. These peculiarities fully justified the long list of names, designating many different diseases—as hysteria, chorea, hydrophobia and tetanus. They all depended upon the reflection of nerve power from the central axis; yet who, on entering a patient's apartment, would fail to detect any of these aberrations of nerve force from the yet more common convulsions dependent upon gastric and intestinal irritation? Depending on a common series of spinal ganglia for their development, does not make them identical, nor even of the same import as to prognosis; for, under the influence of precisely the same *causes*, results of very different significance present themselves, where it is impossible to ascribe the favorableness or unfavorableness of the issue, the danger or safety in the case, to *degree* any more than to *kind*. They all constitute different *kinds* of reflex action. Hysteria is one form, tetanus another. He could not admit that one was essentially an organic disease and the other a functional. Pathology revealed no constant, nor even frequent, change in the cord or its membranes. [Surgeon MCCAW, it may be too minute to be discovered.] I do not admit that pathology which the microscope cannot detect. In some instances, meningeal inflammation had been noticed; but much more frequently no morbid trace whatsoever could be discovered, and this circumstance led many to suppose that the disease depended upon simple hyperæmia of the neurilemma of the notochord, untraceable in the necropsy. Nor could he see any real evidence of the identity between the spasms sometimes noticed in injured limbs, or in a special group of muscles in the limb, consisting in alternate quick and jerking contractions and rapid relaxations, and true tetanus. Besides, it should be remembered that muscular irritability disclosed itself in two very distinct phenomena, described as *clonic* and *tonic* contractions. In tetanus, the contractions were of the tonic variety; slow and long-continued decurtations of the muscular fibres, with gradual relaxations; but, in the jerkings and twitchings of groups of muscles referred to, the muscular contractions were rapid and frequent, and more of the clonic order.

Surgeon BROCK recounted the case of a man whose leg was crushed by the wheel of a car, and amputated a few hours after. The wound soon presented an unhealthy appearance and sloughed extensively, when, twelve days after sloughing commenced, tetanus supervened. The trismus, opisthotonos and general rigidity of the whole muscular system were very marked; constipation and anorexia were among the prominent symptoms; treated with purgatives and opiates in large doses, he recovered. One other case of tetanus was transferred to another hospital, and one died the day after admission.

Surgeon CHAMBLISS related the case of a boy who injured his back by falling from a fence, in whom the spasms commenced on the seventh day, first in legs and thighs, though he was able to walk about until general tetanus was developed, of which he died about seven days after.

Surgeon BOLTON stated that it had been his misfortune to meet with many cases of tetanus, all of which had proved fatal with the single exception of that related by Dr. BROCK. Of these, one was a case of idiopathic tetanus, which occurred under circumstances which forbid the possibility of its having been occasioned by injury or exposure. The patient was an inmate of the Virginia penitentiary. No cause could be discovered for the attack. It resulted fatally. He thought there was a difference between the spasms of true tetanus and tetanic or tetanoid; those resembling the spasms of tetanus, for example, the tetanic spasms of hysteria. He agreed with Dr. MICHEL in the distinction which he drew between tonic and clonic spasms, and by attention to this he believed the diagnosis could usually be made between the spasms of tetanus and those of other diseases. He called attention to another important element of diagnosis, viz: the constitutional symptoms. The pulse becomes full and strong, and the temperature of the skin increased. When the constitutional symptoms set in, no local treatment will be of any avail; even amputation has rarely been of use; and if the entire ablation of the injured part be insufficient to remove the local cause, it is not probable that hypodermic injections at the point of the injured nerve will prove more efficacious.

The hour having expired for the evening's session, the further discussion of the subject was referred to the next meeting, and the Association adjourned.

